# CO_2_ flux from farmland across salinization gradients during freeze–thaw periods under winter irrigation

**DOI:** 10.3389/fpls.2025.1690189

**Published:** 2025-11-07

**Authors:** Fan Luo, Xinghong He, Yiwei Chen, Rui Gao, Yuan Ma, Shiyuan Liu, Yao Guan, Yuying Ma

**Affiliations:** 1 College of Hydraulic and Architectural Engineering, Tarim University, Xinjiang, China; 2 College of Water Conservancy Engineering, Tianjin Agricultural University, Tianjin, China

**Keywords:** seasonal frozen soil, CO_2_ emissions, winter irrigation, salinized farmland, salinity gradient

## Abstract

**Introduction:**

Winter irrigation, as an effective agricultural practice, exerts positive effects on spring-sown crops and is widely applied in Xinjiang, China. Under the influence of seasonal freeze–thaw cycles, the mechanisms by which winter irrigation affects farmland carbon emissions are of great significance for both agricultural production and greenhouse gas emissions. Therefore, conducting relevant research is extremely necessary.

**Methods:**

A field plot experiment was conducted with three salinity gradient levels. The flood irrigation and drip irrigation were applied during the non-growing period following cotton harvest, with three irrigation amounts.

**Results and discussion:**

The results indicated that as the soil froze and thawed, CO_2_ emissions exhibited a trend of initially decreasing and then increasing. During the pre-freezing period, winter irrigation intensified salt accumulation in the unfrozen zones, thereby restricting gas emissions. The rate of decline in CO_2_ fluxes increased with irrigation amount, and this effect became more pronounced as soil salinity increased. In the high- and medium-salinity treatments, irrigation significantly reduced CO_2_ emissions, with the emissions under the irrigation treatments being approximately half of those observed in the control treatment. However, during the thawing period, the redistribution of soil salt and moisture weakened the effect of irrigation and irrigation no longer had no significant effect on CO_2_ emissions. The soil salinity became the only influential factor. Moreover, since CO_2_ emissions during the thawing period were much higher than those during the pre-freezing period, the overall effect of winter irrigation on CO_2_ emissions across the entire freeze–thaw cycle was not significant. From the perspective of carbon sequestration and emission reduction, winter irrigation is a neutral agricultural practice, neither reducing carbon emissions nor increasing the risk of carbon release.

## 1 Introduction

Carbon emissions, as a key pathway of soil organic carbon consumption, are pivotal not only for maintaining agricultural productivity but also for driving global climate change (Lal, 2003; Wu et al., 2025). Located in arid and semi-arid regions, Xinjiang experiences uneven soil moisture distribution and strong temperature fluctuations during spring and autumn, leading to seasonal frozen soil. The transformation between liquid and solid phases of water during freeze–thaw cycle leads to the breakdown of soil aggregates (Liu et al., 2023a), which can modify soil porosity and influence the efficiency of gas exchange (Han et al., 2018; Sun et al., 2021). In addition, freeze–thaw processes could modify the redistribution of soil water and salts (Yang and Wang, 2019; Liu et al., 2022a), and disturb soil microbial activity and community structure (Skogland et al., 1988; Han et al., 2018). The individual alterations of these factors, as well as their combined interactions, can influence soil carbon emissions from farmlands (Snyder et al., 2009; Wu et al., 2014; Han et al., 2018; Yang et al., 2023b, Yang et al., 2023d). Moreover, under freeze-thaw conditions, variations in soil temperature can affect water soil water-salt regulation but also affect enzyme activity (Liu et al., 2017), thereby impacting the carbon emission dynamics (Wu and Shen, 2010; Meijide et al., 2017; Yang et al., 2023c; Li et al., 2025a). Under the unique climatic conditions of Xinjiang, freeze-thaw action have a particularly significant effect on soil carbon emissions. Therefore, understanding the impact of freeze-thaw action on soil carbon emissions is essential, not only for maintaining soil health and crop productivity but also for mitigating global greenhouse gas emissions.

Moreover, salinity in the soil can influence the activity of certain microorganisms, and alter the volume of organic matter decomposition (Wichern et al., 2006; Rath and Rousk, 2015). The high salinity reduces the osmotic potential of the soil solution, thereby inhibiting microbial activity and slowing the decomposition of organic matter, which ultimately affects CO_2_ emissions (Yuan et al., 2007; Haj-Amor et al., 2022). However, in certain regions of Xinjiang, soil salinization is widespread. Elevated salt concentrations may deteriorate soil structure, affect freeze-thaw processes, and consequently change soil water movement dynamics (Fu et al., 2018; Liu et al., 2022a, Liu et al., 2023b; Tan et al., 2023; Yu et al., 2024; Zhang et al., 2024). The Xinjiang region is characterized by extensive and severe soil salinization. Effective control of irrigation water input is not only essential for the remediation of saline–alkali soils, but also critical for regulating soil moisture and thermal regimes during freeze-thaw cycles (Kurganova et al., 2007).

Following the autumn harvest in Xinjiang, the flood irrigation is implemented in agricultural fields (He et al., 2016; Qian et al., 2021). This conventional approach to saline-alkali land management facilitates salt leaching into deeper soil profiles, which helps to maintain favorable germination rates for crops sown in the subsequent spring (Liu et al., 2022b). Moreover, it contributes to the replenishment of deep soil moisture. This non-growing season irrigation, known as winter irrigation, involves a single application of water that exceeds one-third of the total annual irrigation quota (Wu et al., 2020). While winter irrigation serves as an effective approach to ameliorating saline–alkali soils, flood irrigation practices may elevate the risk of soil erosion (Cerdà et al., 2021), reduce soil quality, and ultimately threaten food security. In addition, flood irrigation can damage the surface soil structure, resulting in non-uniform water infiltration. With the widespread adoption of drip irrigation technology, some regions in Xinjiang have begun using drip irrigation for winter irrigation (Feng et al, 2021). Compared with traditional flood irrigation, drip irrigation offers substantially improved controllability and precision (Hou et al., 2025). Irrigation methods have been shown to significantly influence soil water distribution and gas exchange volumes (Linn and Doran, 1984; Wong et al., 2010; Chen et al., 2016; Yang et al., 2023a). Moderate irrigation can alleviate water and salt stress, enhance soil gas exchange efficiency (Chen et al., 2015; Hou et al., 2019; Wang et al., 2019; Gao et al., 2021). The fluctuations in soil moisture may affect microbial community activity, altering the decomposition of organic matter (Wang and Xu, 2018; Min et al., 2023). Moreover, different irrigation methods result in varying soil desalination efficiencies, which in turn cause heterogeneity in salt distribution (Wang et al., 2024). This could potentially affect microbial activity and, in turn, alter carbon emission processes in agricultural soils (Wei et al., 2016; Liang et al., 2021).

From the perspective of agricultural production, the magnitude and rate of carbon emissions serve as key indicators of the carbon balance in agricultural production systems (Lockhart et al., 2023). Carbon dioxide (CO_2_) and methane (CH_4_) are the predominant pathways of soil carbon emissions (Wu et al., 2014; Yang et al., 2023b). They are strongly influenced by oxygen availability (Min et al., 2023; Arunrat et al., 2025). Under well-aerated conditions in dryland fields, carbon loss from farmland occurred primarily in the form of CO_2_. Although many studies have examined the effects of irrigation, freeze–thaw processes, and soil salinity on CO_2_ emissions from farmland (Badewa et al., 2022; Haj-Amor et al., 2022; Liu et al., 2022a), research on their combined effects remains limited. Under the unique climatic conditions of Xinjiang, the effect of winter irrigation on water and salt distribution in farmland may lead to soil carbon emission patterns that differ from those during non-freeze–thaw periods. Given the scarcity of related studies in Xinjiang, it is of great significance to investigate the effect of winter irrigation on carbon emissions during freeze–thaw cycles in this region. In order to understand the influence of winter irrigation on CO_2_ emissions from salinized farmland subjected to freeze-thaw action, the following researches were carried out: i) to measure the CO_2_ emission processes under different experimental conditions; ii) to analyze the effects of seasonal freeze–thaw cycles, winter irrigation, and soil salinity on CO_2_ emissions.

## 2 Method and materials

### 2.1 Experimental area

**Figure 1:**
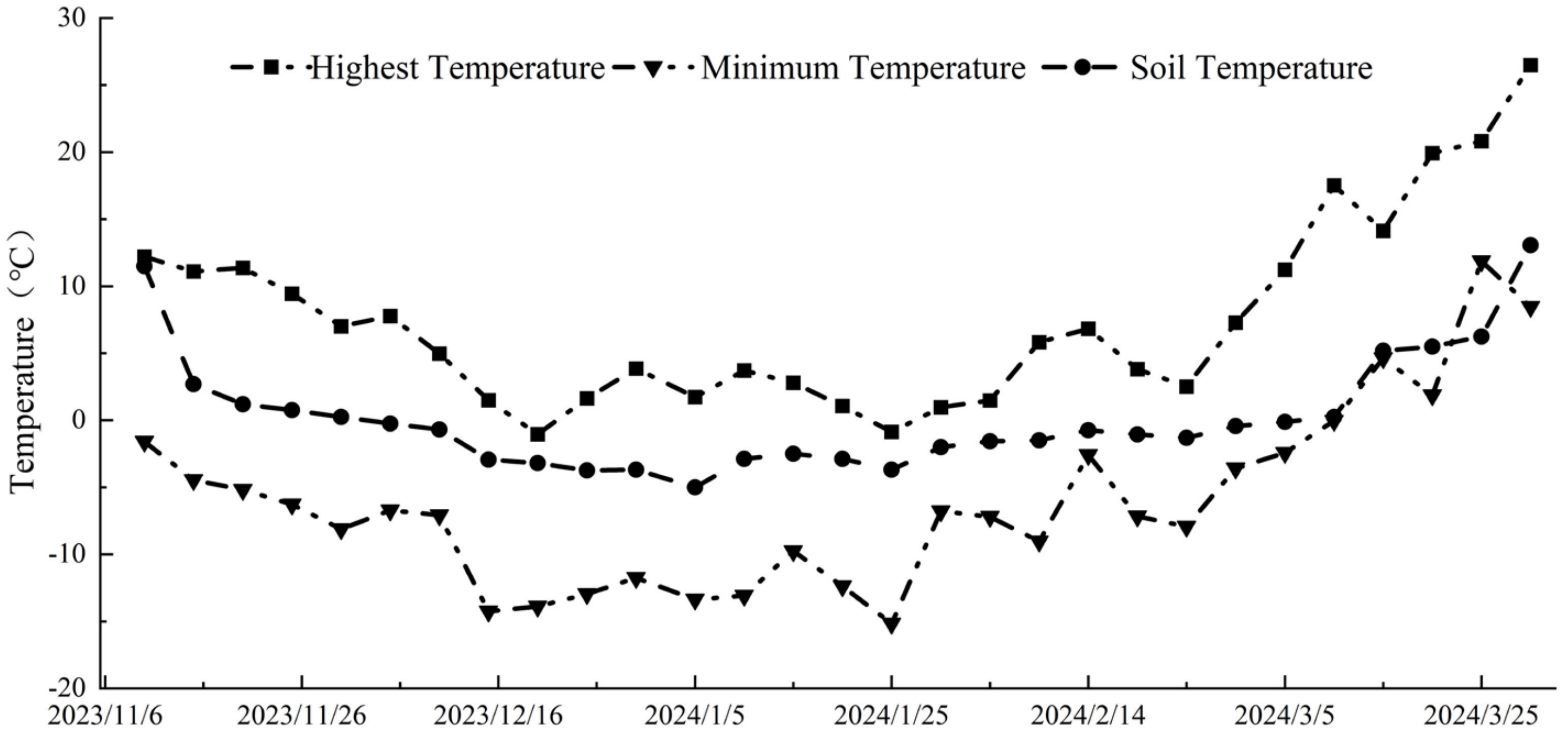
Soil and air temperature trends during seasonal freezing and thawing.

The experiment was conducted at the water-saving irrigation experimental base of the College of Hydraulic and Architectural Engineering, Tarim University, located in Aral City, Xinjiang Uygur Autonomous Region, China. The experimental area is situated at 81°17′ E longitude and 40°32′ N latitude, and it has a temperate continental climate characterized by low precipitation, and strong surface evaporation. The average annual precipitation ranges from 40.1 to 82.5 mm, while the average annual evaporation ranges from 1876.6 to 2558.9 mm. The multi-year average temperature in winter is -4.1°C, with an extreme low of -15°C. The region undergoes seasonal soil freezing but is not characterized by permafrost. **Figure 1** illustrates the variations in soil and air temperatures during the freeze-thaw period.

### 2.2 Soil characteristics

The soils in the study area were classified as clay loam, composed of 33.4% clay (< 0.002 mm), 44.2% silt (0.002 to 0.05 mm), and 22.4% sand (0.05 to 2 mm). The bulk density of the farmland soil was measured *in situ* using a cutting ring, and the value was determined to be 1.35 g cm^-3^. The field capacity of the soil in experimental farmland was 28.4% (v/v). Additionally, the soil pH was approximately 8.3 and the content of soil organic carbon was 14.28 g kg^-1^. Before irrigation, soil samples were collected from different layers (0-20, 20-40, 40–60 cm) in the experimental field, and the average electrical conductivity of each soil layer was subsequently determined. Soil solutions were prepared at a soil-to-distilled water ratio of 1:5, with the mixtures thoroughly shaken and then allowed to stand. The average electrical conductivity (EC_1:5_) of each soil layer was subsequently determined using the supernatant. The experimental area was divided into three salinity gradients: T_1_ (2.0-3.0 dS m^-1^, low salinity), T_2_ (3.0-3.9 dS m^-1^, medium salinity), and T_3_ (4.5-6.5 dS m^-1^, high salinity).

### 2.3 Experimental methods

In this study, after the cotton was harvested in the experimental field, winter irrigation was carried out using two irrigation methods. The irrigation water used in the experiment was tap water processed by the water treatment plant, which avoided the introduction of excessive additional salts. Alongside traditional flood irrigation, drip irrigation with enhanced water management capabilities was implemented, namely drip irrigation and flood irrigation. The irrigation amounts were set at three levels: 0, 120 and 240 mm. Among them, the 0 mm treatment served as the blank control. Among them, W_3_ represents the commonly used water amount for winter irrigation. Each experimental plot measured 4 m × 4 m, with a total area of 16 m^2^. Plots were separated by plastic film buried to a depth of 60 cm, and earthen ridges were constructed aboveground. Drip irrigation was implemented using 40 mm-diameter PVC drip pipes, each connected to three drip tapes within each plot. To minimize the influence of spatial heterogeneity, the experiment was conducted in a randomized complete block design with three replications per treatment, evenly distributed along the soil salinity gradient. All plots were situated at a uniform elevation and shared the same diluvial parent material. The experimental treatments are summarized in **Table 1**.

**Table 1:** Abbreviations of each experimental treatment.

Salinity gradient		Irrigation method		Irrigation amount	
T_1_	Low	G_1_	Flood irrigation	W_1_	0
T_2_	Medium	G_2_	Drip irrigation	W_2_	120 mm
T_3_	High			W_3_	240 mm

### 2.4 Data collection and analysis

Data were collected from early November 2023 to early April 2024 using the static chamber-gas chromatography method. After cotton harvesting was completed in late October 2023, stainless steel bases (50 cm × 50 cm × 20 cm) with a 1 cm deep and 1 cm wide groove on the top edge were installed in experimental zone. During sampling, a stainless-steel chamber (50 cm × 50 cm × 50 cm) was placed into the groove, and the groove was filled with water to ensure an airtight seal. The outer surface of the chamber was wrapped with aluminum foil and sponge to minimize the influence of ambient temperature on internal gas concentration. Each chamber was equipped with a small fan on the top to ensure uniform air mixing during sampling, and a thermometer to monitor and record the internal temperature.

Gas sampling was carried out from the pre-freezing to the thawing period, between 11:00 a.m. and 1:00 p.m. on each sampling day. At each sampling event, the gas was extracted using a 50 mL syringe and injected into an aluminum foil gas sampling bag equipped with a single-metal valve. Subsequent gas samples were collected at 10-minute intervals, for a total of six samples per event. After each sampling event, all collected gas samples were promptly brought back to the laboratory for analysis using a gas chromatograph. The gas emission fluxes were calculated using Equation 1.

(1) $$F=\rho \text{h}\frac{273}{273+T}\times \frac{\Delta \text{c}}{\Delta t}$$

where *F* is the gas emission flux, mg·m^-2^·h^-1^; *ρ* is the gas density under standard conditions, mg·m^-3^; *h* is the chamber height, m; *T* is the average centigrade temperature inside the chamber; Δc/Δt is the rate of change in gas concentration during the sampling period.

### 2.5 Division of the experimental period

Based on historical meteorological data from the Aral City of Xinjiang, the pre-freezing period was defined as the interval from the first occurrence of daily minimum temperatures falling below 0°C to the point when daily maximum temperatures remained below 0°C (November 16 to December 12, 2023). The thawing period was defined as the interval from the first occurrence of daily maximum temperatures rising above 0°C to the complete thawing of the soil (February 17 to April 1, 2024). The interval between the pre-freezing period and the thawing period is referred to as the freezing period (December 13, 2023 to February 16, 2024). Continuous monitoring of gas emissions was conducted during the pre-freezing period and the thawing period, with soil CO_2_ flux measured every three days. However, no measurements were conducted during the freezing period.

### 2.6 Measurement and analysis

Soil moisture and temperature were measured during the entire freeze–thaw period. At each measurement event, the mean soil moisture and temperature within the top 20 cm of the soil profile were measured using soil moisture and temperature sensors. The soil moisture sensors are based on conductivity conversion. To minimize the impact of soil salinity on measurement accuracy, the sensors were calibrated in the laboratory before being installed at the predetermined measurement sites. In addition, soil moisture content was expressed as volumetric water content. Meteorological data for the experimental area were provided by the Aral Meteorological Bureau.

### 2.7 Data processing and statistical analysis

Statistical significance was assessed using SPSS 26.0, and differences among treatments were evaluated using the least significant difference (LSD) method at a significance level of P<0.05. Pearson correlation analysis was employed to examine the relationships between cumulative CO_2_ emission fluxes and environmental factors. All figures were generated using Origin 2022.

## 3 Results

### 3.1 Soil liquid water content during seasonal freeze-thaw action

**Figure 2:**
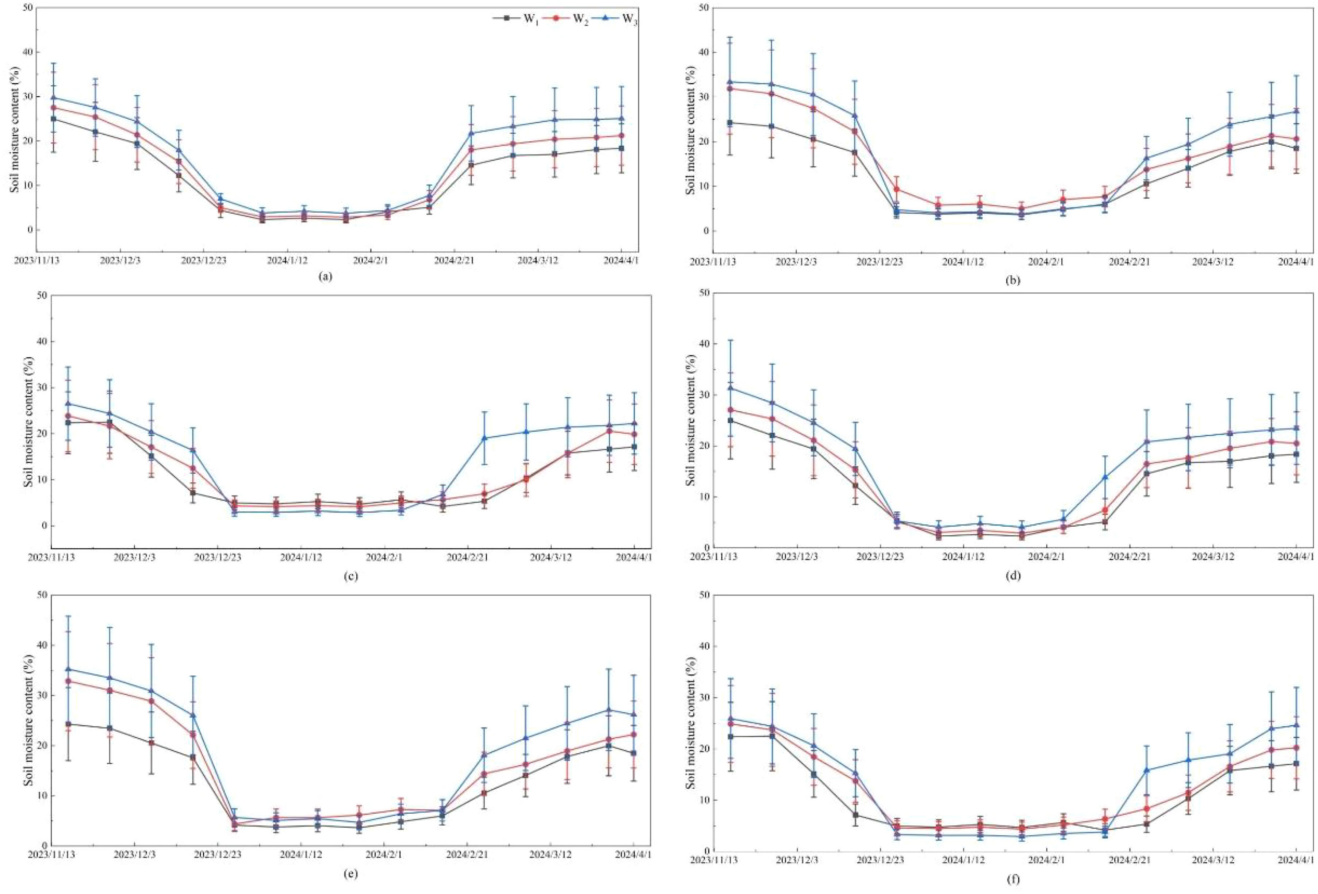
Measured soil liquid water content during the freeze–thaw period. **(a)** G_1_T_1_, **(b)** G_1_T_2_, **(c)** G_1_T_3_, **(d)** G_2_T_1_, **(e)** G_2_T_2_, **(f)** G_2_T_3_.

During seasonal freeze–thaw periods, soil water typically existed in both liquid and frozen forms. In this study, the soil moisture recorded by the sensors was more indicative of the liquid water content within the soil, which did not exceed the actual soil moisture content. The measurement results were shown in **Figure 2**. It should be noted that during pre-freezing period, liquid water gradually transformed into solid ice. As air temperature decreased and soil freezing progressed, the liquid water content steadily declined. By the freezing period, the soil water had largely transitioned into the frozen state and the liquid water content dropped to a very low level. Subsequently, as the air temperature rose, the frozen water gradually melted, and the liquid water content increased progressively. By late March to early April, the frozen soil had completely thawed, and the liquid water content reached its maximum value. At this point, the frozen soil water completely melted, converting into liquid water. Overall, the measured liquid water content exhibited a trend consistent with soil temperature fluctuations and was strongly influenced by freeze–thaw processes.

### 3.2 Soil CO_2_ emission variation under different experimental conditions

#### 3.2.1 The pre-freezing period

**Figure 3:**
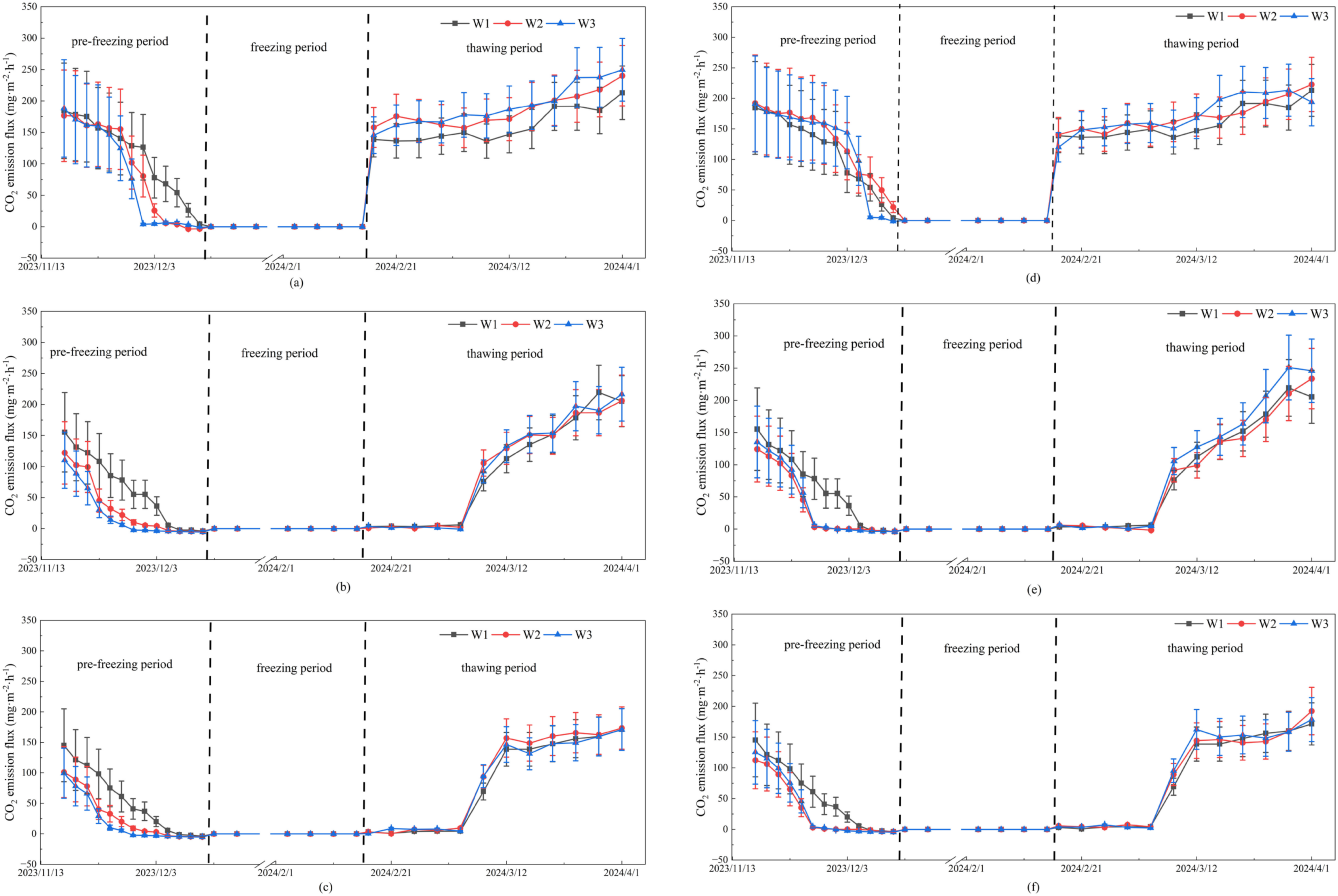
Measured CO_2_ emission flux during the freeze-thaw period. **(a)** G_1_T_1_, **(b)** G_1_T_2_, **(c)** G_1_T_3_, **(d)** G_2_T_1_, **(e)** G_2_T_2_, **(f)** G_2_T_3_.

During the pre-freezing period, the CO_2_ emission flux showed a declining trend over time, with the rate of decline accelerating as irrigation amount increased (**Figure 3**). The W_1_ treatment experienced the slowest decrease in CO_2_ flux, requiring the longest duration to reach the minimum emission level. The CO_2_ fluxes were strongly influenced by irrigation amount in the T_2_ and T_3_ treatments, whereas this effect was relatively minor in the T_1_ treatment. However, under irrigation treatments, no significant differences were observed in the CO_2_ fluxes between different irrigation methods or irrigation amounts. In addition, throughout the entire pre-freezing period, the CO_2_ flux in the T_1_ treatment was substantially higher than those in the T_2_ and T_3_ treatments.

**Figure 4:**
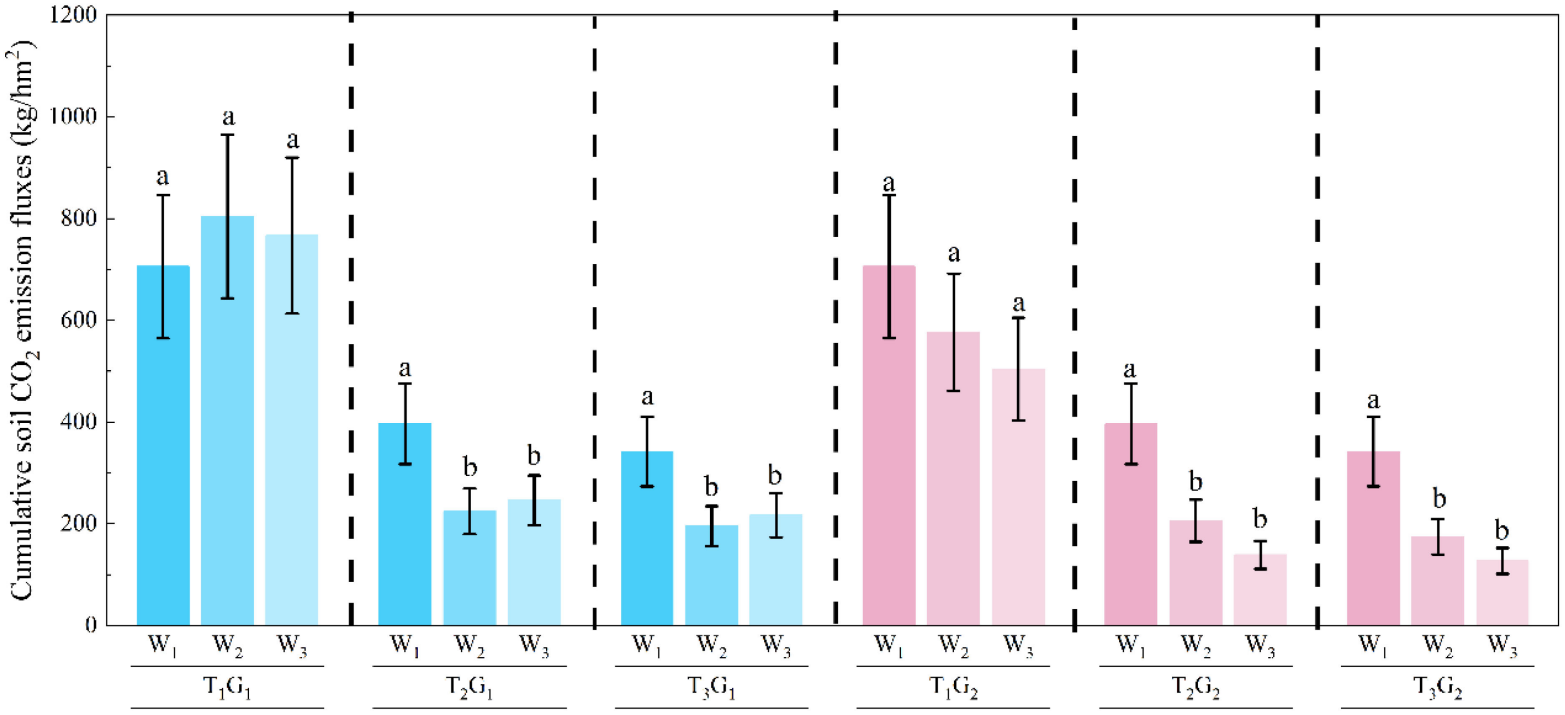
Cumulative CO_2_ emissions during the pre-freezing period from Nov. 16 to Dec. 12, 2023. The different lowercase letters indicated significant differences at the p < 0.05 level.

The cumulative CO_2_ emissions during the pre-freezing period were shown in **Figure 4**. For the T_1_ treatment, the cumulative CO_2_ emissions under the W_1_, G_1_W_2_, G_1_W_3_, G_2_W_2_ and G_2_W_3_ treatments were 799.2, 924.5, 893.3, 924.5 and 893.3 kg·hm^-2^, respectively. Similarly, for the T_2_ treatment, the cumulative CO_2_ emissions were 396.7, 224.5, 198.0, 205.9 and 138.9 kg·hm^-2^, respectively. For the T_3_ treatment, the cumulative CO_2_ emissions were 341.8, 195.5, 216.9, 174.8 and 126.9 kg·hm^-2^, respectively. The results indicated that CO_2_ emissions increased as soil salinity decreased, and that high salinity exerted a strong suppressive effect on carbon emissions from farmland soils. Moreover, for both T_1_ and T_2_ conditions, cumulative CO_2_ emissions were significantly reduced in W_2_ and W_3_ compared to W_1_, suggesting that winter irrigation played a role in mitigating CO_2_ release from the farmland with high salinity. However, for the T_1_ treatment, cumulative CO_2_ emissions did not differ significantly between irrigated and non-irrigated plots, indicating that winter irrigation had no effect on CO_2_ emissions from the farmland with low salinity.

#### 3.2.2 The thawing period

The **Figure 3** illustrated the dynamic changes in CO_2_ fluxes throughout the freezing period. With increasing temperature and the gradual thawing of the soil, CO_2_ was gradually released from all experimental plots. The onset time of CO_2_ release under the T_1_ treatment was earlier than those under the other two treatments. Consequently, in the initial phase of soil thawing, the CO_2_ fluxes in the T_1_ treatment were higher than those in the T_2_ and T_3_ treatments. Once the CO_2_ emissions began in the T_2_ and T_3_ plots, their emission fluxes increased rapidly, narrowing the difference with the T_1_ treatment. However, no notable differences in CO_2_ fluxes were observed among the different irrigation amounts. This indicated that autumn irrigation did not affect CO_2_ fluxes from the farmland during the thawing period.

**Figure 5:**
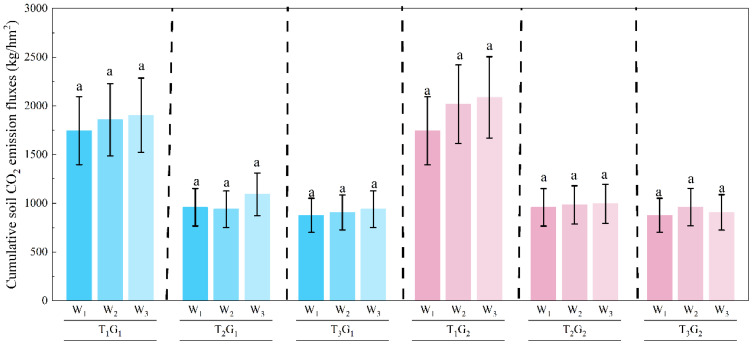
Cumulative CO_2_ emissions during the thawing period from Dec. 13, 2023 to Feb. 16, 2024. The different lowercase letters indicated significant differences at the p < 0.05 level.

During the thawing period, CO_2_ emissions increased significantly as the soil thawed. Additionally, a close relationship was observed between CO_2_ emissions and soil salinity. Before early March, the CO_2_ emission flux from the T_1_ treatment were higher than those from the other two treatments. From early March to mid-March, the differences in CO_2_ emission flux across different salinity treatments were rapidly diminishing. In the final stage of the freezing period, CO_2_ fluxes across the three soil salinity gradients showed only minor differences. Furthermore, the cumulative CO_2_ emissions throughout the thawing period in the T_1_ treatment were much greater than those observed in the T_2_ and T_3_ treatments (**Figure 5**). However, there was no significant difference in cumulative CO_2_ emissions between T_1_ and T_2_. Considering the changes in CO_2_ emission fluxes during the freezing period, it was evident that the combined effects of freeze–thaw cycles and soil salinity exerted a substantial influence on gas emission processes. Additionally, neither the irrigation amount nor the irrigation method significantly affected gas emissions during this stage.

### 3.3 Cumulative CO_2_ emissions over the entire soil freeze-thaw period

**Figure 6:**
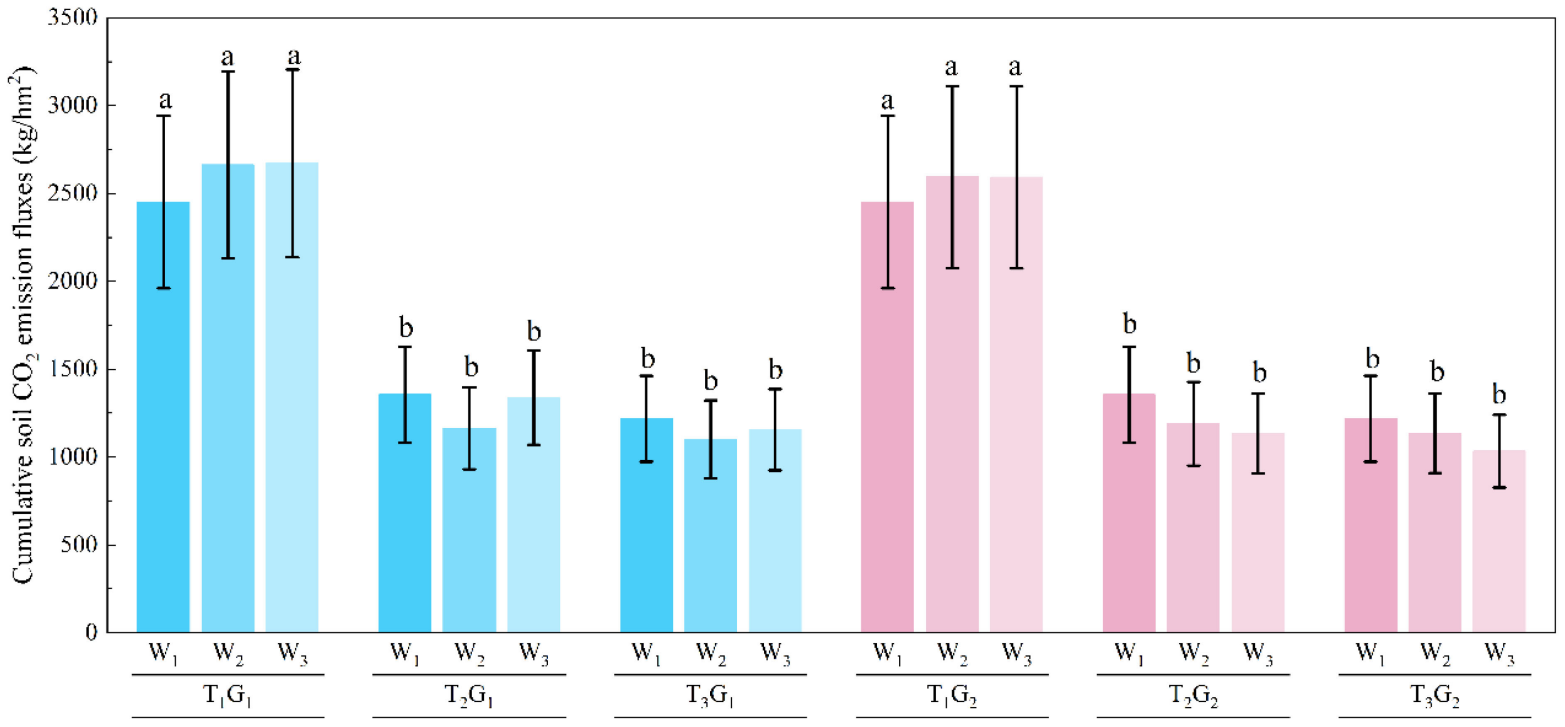
Cumulative CO_2_ emission over the entire soil freeze–thaw period. The different lowercase letters indicated significant differences at the p < 0.05 level.

The cumulative CO_2_ emissions over the entire soil freeze-thaw period under different experimental conditions were shown in **Figure 6**. The results indicated that soil salinity significantly influenced the cumulative CO_2_ emissions over the freeze-thaw period, while neither irrigation method nor irrigation amounts had a significant impact. For the T_3_ treatment, cumulative CO_2_ emissions ranged from 1009.0 to 1203.1 kg·hm^-2^ under different irrigation methods and volumes. For T_2_, cumulative CO_2_ emissions ranged from 1094.5 to 1340.5 kg·hm^-2^. In comparison, the T_1_ treatment showed the highest cumulative CO_2_ emissions, ranging from 2592.2 to 2845.7 kg·hm^-2^ under the same irrigation conditions. The cumulative CO_2_ emissions under the T_1_ treatment were approximately 2.5 times higher than those under the T_2_ or T_3_ treatments. From T_1_ to T_2_, CO_2_ emissions decreased with increasing soil salinity, whereas no significant difference was observed between T_2_ and T_3_. This observation suggests a potential threshold between T_1_ and T_2_, but verification requires experiments with more salinity gradients. Considering solely the objective of carbon sequestration and emission mitigation over the freeze-thaw period, high soil salinity was advantageous for farmland.

As shown in **Table 2**, cumulative CO_2_ emission fluxes were positively correlated with soil liquid water content, with correlation coefficients ranging from 0.035 to 0.852. A similar positive correlation was observed between CO_2_ emissions and soil liquid water content, with coefficients ranging from 0.395 to 0.853. The soil liquid water content was strongly influenced by freeze–thaw processes, decreasing progressively as the soil froze and increasing again as the soil thawed. Therefore, soil liquid water content and soil temperature exhibited synchronous variations during the freeze–thaw process. Their effects on CO_2_ emission dynamics were consistent, both contributing to the observed seasonal pattern of CO_2_ fluxes. Considering that soil temperature could serve as the driving factor for changes in soil liquid water content and was more readily measurable, it might be regarded as the main factor influencing CO_2_ emission fluxes.

**Table 2:** Correlation coefficients between soil CO_2_ emissions and soil temperature and soil liquid water content under different treatments.

Treatment	Water content	Soil liquid water content
T_1_W_1_	0.852**	0.649**
T_1_G_1_W_2_	0.431*	0.675**
T_1_G_1_W_3_	0.769**	0.712**
T_1_G_2_W_2_	0.603*	0.719**
T_1_G_2_W_3_	0.036	0.797**
T_2_W_1_	0.815**	0.556
T_2_G_1_W_2_	0.723**	0.448
T_2_G_1_W_3_	0.657*	0.408
T_2_G_2_W_2_	0.572	0.636*
T_2_G_2_W_3_	0.288	0.395
T_3_W_1_	0.035	0.777**
T_3_G_1_W_2_	0.634*	0.729**
T_3_G_1_W_3_	0.682*	0.767**
T_3_G_2_W_2_	0.784**	0.853**
T_3_G_2_W_3_	0.208	0.803**

** indicates significance at the 0.01 level, and * indicates significance at the 0.05 level.

## 4 Discussions

### 4.1 Impact of winter irrigation on soil CO_2_ emissions

Soil water is an essential condition for microbial activity. Irrigation amount and method significantly influence soil physical properties, microbial community structure, and their physiological activities by regulating soil moisture and thermal conditions (Marton et al., 2012; Kontopoulou et al., 2015; Hou et al., 2019; Yang et al., 2023a), thereby altering the soil CO_2_ emission processes (Skogland et al., 1988; Wang et al., 2019). During non-freeze–thaw periods, increasing soil moisture through irrigation can promote CO_2_ emissions from the soil (Zou et al., 2014; Chi et al., 2017; Yang et al., 2023c). Nevertheless, it is noteworthy that following winter irrigation, ambient air temperature steadily declined, eventually dropping below 0 °C. The surface of irrigated plots was more prone to ice formation because their higher surface soil moisture, compared to non-irrigated plots, promoted ice crystallization (Boike et al., 1998; Wu et al., 2017). The developed ice crystals tend to seal surface pores, restricting gas exchange. Furthermore, during the freezing process, pure water in saline soil freezes first, causing salts to concentrate in the remaining unfrozen liquid water (Liu et al., 2022a; Yu et al., 2024). Although irrigation can leach most surface salts into deeper soil layers, salt accumulation in the unfrozen water still leads to the formation of high salinity zones in the surface soil. The localized secondary salinization within soil can reduce osmotic potential, inhibiting microbial growth and enzyme activity, which in turn suppresses organic matter decomposition and CO_2_ emissions (Rath and Rousk, 2015; Yu et al., 2020; Haj-Amor et al., 2022). Therefore, CO_2_ emission fluxes in the irrigated plots decreased to minimal levels more rapidly compared to the non-irrigated plots (**Figure 4**). Overall, salt leaching through irrigation reduced surface soil salinity and thereby promoted CO_2_ emissions; however, irrigation also intensified the freezing of surface soil water, leading to localized secondary salinization that inhibited CO_2_ emissions. The combined effects of these two driving factors determined the soil gas emissions in farmland during the pre-freezing period. For the T_2_ and T_3_ treatments, the inhibitory effect of localized secondary salinization was dominant, resulting in significantly lower CO_2_ emissions in the irrigated plots compared to the non-irrigated controls. For the low-salinity T_1_ treatment, the promoting effect of salt leaching was counteracted by the inhibitory effect of localized secondary salinization, resulting in no significant difference in cumulative CO_2_ emissions between the irrigated and non-irrigated plots.

In this study, only a single irrigation event was conducted before the freeze–thaw cycles, with no further irrigation applied thereafter. The freeze–thaw cycles can facilitate the upward migration of soil moisture and salts from deeper layers to the surface (Wang et al., 2023; Luo et al., 2024). The upward translocation of salts reduces the effectiveness of irrigation-induced leaching. Consequently, the irrigation did not have a significant effect on CO_2_ emissions during the thawing phase. Overall, irrigation affected soil CO_2_ fluxes markedly before full soil freezing, but showed a gradually diminishing influence during soil thawing. The water volume used in winter irrigation is substantially greater than the irrigation quota for a single application in the growing season. Despite the large differences in irrigation amounts applied in this study, both treatments can be classified as excessive irrigation. Both irrigation amounts elevate the soil moisture content within a certain depth of the surface layer to near saturation. Therefore, during the soil freezing process, the localized secondary salinization in the soil exhibited minimal differences between the two irrigation amounts. The irrigation amounts applied have no significant effect on CO_2_ emissions throughout the freeze–thaw process.

### 4.2 Soil CO_2_ emission trends during the freeze-thaw period

The influence of temperature changes within freeze–thaw periods on soil CO_2_ emissions is significant and warrants attention. Soil CO_2_ emissions primarily originate from soil respiration and microbial decomposition processes (Lei et al., 2021). As temperatures rise, soil respiration intensifies, resulting in a notable increase in cumulative CO_2_ emissions; conversely, lower temperatures restrict CO_2_ emissions. Correlation analysis in our study further confirmed a positive relationship between soil temperature and cumulative CO_2_ emissions, indicating the key regulatory role of temperature in regulating CO_2_ fluxes dynamics, consistent with previous findings (Ray et al., 2020; Kitamura et al., 2021).

During the freezing period, soil temperature reaches its minimum, and the low temperatures restrict microbial activity (Wu and Shen, 2010; Protti-Sánchez et al., 2025). As the soil freezes, the decrease in liquid water content limits the water available for microbial metabolism. Furthermore, ice formation blocks soil pores and surface micro-fissures, thereby impeding gas exchange. Together, these factors substantially reduce CO_2_ emissions (Tilston et al., 2010; Badewa et al., 2022; Francioni et al., 2025). Across the full freeze–thaw cycle, CO_2_ emissions in the freezing period remained far lower than those observed during the pre-freezing and thawing periods. Although CO_2_ emissions in the freezing period were excluded from the cumulative total, their effect on the overall results is negligible.

In this study, cumulative CO_2_ emissions during the thawing period were significantly higher than those observed during the pre-freezing period, aligning with the findings of Wagner and Pfeiffer (1997), who emphasized that the content and mineralization rate of soil carbon and nitrogen substrates govern CO_2_ emission intensity. Moreover, Sharma et al. (2006) reported that freeze-thaw processes can disrupt soil aggregates, releasing large amounts of labile organic carbon for microbial utilization and accelerating organic matter mineralization. Physical damage to frozen soils during the freezing period can cause root cell rupture and death, providing a substantial carbon source, which likely accounts for the higher CO_2_ emissions during thawing. Furthermore, Herrmann and Witter (2002) demonstrated that freeze-thaw processes not only lead to microbial cell death but also increase the availability of nutrients such as carbon, supplying surviving microorganisms with abundant substrates and thereby further promoting CO_2_ emissions. As observed in previous studies, cumulative soil CO_2_ emission fluxes remained low before soil freezing and exhibited a sharp increase during the thawing period.

Freeze–thaw cycles modify the dynamics of water, salt, and heat movement in soils (Qin et al., 2021; Wang et al., 2022; Li et al., 2025b), which can affect soil CO_2_ emissions. This largely results in CO_2_ emission mechanisms that differ from those during non-freeze–thaw periods. For instance, in the freezing phase, water and salts migrate upward toward the freezing front, causing surface salt accumulation and widening the high-salinity zone. In the thawing phase, a secondary upward migration peak further modifies the soil salinity distribution. In addition, soils with different salinity levels also exhibited distinct behaviors during freeze–thaw cycles. In treatments T_2_ and T_3_, elevated soil salinity resulted in a depressed freezing point and slower freezing rates (Amankwah et al., 2021). Nevertheless, salt exclusion from the frozen zone was more pronounced, thereby increasing salinity in the unfrozen soil (Baker and Osterkamp, 1989). As a result, the differences in soil salinity profiles between these treatments and T_1_ became more pronounced. These disparities gradually decreased during the soil thawing process. Furthermore, as temperature increases, the sensitivity of CO_2_ emissions to soil salinity decreases (Yu et al., 2020). For this reason, the CO_2_ fluxes in T_2_ and T_3_ remained lower than those in T_1_ until complete soil thawing, after which no significant differences in CO_2_ fluxes were observed among the salinity gradients. It is not appropriate to assess the influences of temperature, soil moisture, and salinity on CO_2_ emissions independently during freeze–thaw periods. These factors should be considered synergistically when assessing their impact on CO_2_ emissions.

Freeze–thaw cycles not only increase the complexity of CO_2_ emission mechanisms but also pose greater challenges for *in situ* field monitoring. For example, existing measurement techniques are not yet capable of accurately distinguishing between frozen and unfrozen water in soils. This substantially constrains the analysis of dynamic water and salt redistribution processes. The spatial heterogeneity of soil properties further increases the complexity of CO_2_ emission mechanisms and increases the difficulty of analyzing results. Moreover, several inherent limitations of this study should be acknowledged. First, the experiment was conducted on a single soil texture, whereas soil texture is known to regulate water and salt redistribution during irrigation leaching and freeze–thaw processes, as well as microbial activity, all of which jointly affect CO_2_ emission dynamics. Future studies should therefore consider multiple soil textures to evaluate their influence on CO_2_ fluxes. Second, the irrigation water used in this study was tap water, while brackish or saline water is often applied in practice. Such water quality differences may alter soil salinity dynamics and CO_2_ emissions. Future research should explicitly address this factor to provide a more comprehensive understanding. Finally, the results were derived from a single experimental year, and thus may not capture interannual variability. Long-term or multi-year monitoring is required to better characterize the variability and trends of gas emissions from saline–alkali soils under freeze–thaw conditions.

## 5 Conclusions

This study analyzed the characteristics of soil CO_2_ emissions from saline-alkaline farmland under different irrigation amounts and methods during freeze-thaw action. The main conclusions are as follows.

During the pre-freezing period, winter irrigation had an influence on CO_2_ emissions, especially in plots with high soil salinity. The CO_2_ emissions from irrigated plots with high salinity were significantly lower than those from non-irrigated plots. However, CO_2_ emissions showed no significant relationship with winter irrigation in low salinity plots. Once thawing began, the differences in soil water and salinity conditions under different irrigation treatments were minimal, resulting in no significant effect of winter irrigation on CO_2_ emissions. In addition, the influence of soil salinity on CO_2_ emissions persisted throughout most of the freeze–thaw period, with CO_2_ emissions under high salinity significantly lower than those under low salinity. Following freeze–thaw cycles, soil water and salt underwent redistribution, resulting in a reduced effect of experimental treatments on CO_2_ fluxes during the later thawing phase. Based on the characteristics of CO_2_ flux and cumulative emissions, there appears to be a potential salinity threshold between the T_1_ and T_2_ treatments.

This study found that under freeze–thaw conditions, winter irrigation only reduced CO_2_ emissions during the freezing period at high salinity levels, and the reduction was limited. Over the full freeze–thaw cycle, winter irrigation had no significant effect on cumulative CO_2_ emissions. Thus, from a carbon emission perspective, winter irrigation has a relatively neutral impact on CO_2_ fluxes, with its primary benefits likely related to soil water–salt regulation and improved cultivation conditions.

## Data Availability

The datasets presented in this article are not readily available because the data presented in this study are available on request from the corresponding author. Requests to access the datasets should be directed to mayuying@tjau.edu.cn.
